# Prognostic relevance of melanoma antigen D1 expression in colorectal carcinoma

**DOI:** 10.1186/1479-5876-10-181

**Published:** 2012-08-31

**Authors:** Zhao-lei Zeng, Wen-jing Wu, Jing Yang, Zhen-jie Tang, Dong-liang Chen, Miao-zhen Qiu, Hui-yan Luo, Zhi-qiang Wang, Ying Jin, De-shen Wang, Rui-hua Xu

**Affiliations:** 1 State Key Laboratory of Oncology in Southern China, Guangzhou 510060, China; 2 Department of Experimental Research, Sun Yat-sen University Cancer Center, Guangzhou, Guangdong, 510060, China; 3 Department of Medical Oncology, Sun Yat-sen University Cancer Center, Guangzhou 510060, China

**Keywords:** MAGED1, Colorectal cancer, Melanoma antigen and prognosis

## Abstract

**Background:**

Melanoma antigen D1 (MAGED1) is a member of the type II melanoma antigen (MAGE) family. The down-regulation of MAGED1 expression has been shown in breast carcinoma cell lines and in glioma stem cells and may play an important role in apoptosis and anti-tumorigenesis. However, there is no report on its clinical role in colorectal cancer (CRC).

**Methods:**

We examined the expression of MAGED1 by qPCR in colorectal cancer tissues and their adjacent non-tumorous tissues taken from 6 cases and performed Western blotting and IHC analyses. In addition, we analyzed MAGED1 expression in 285 clinicopathologically characterized colorectal cancer patients.

**Results:**

MAGED1 expression was significantly down-regulated in colorectal cancer tissues compared with adjacent non-tumorous tissues and was associated with clinical stage (*p* < 0.001), T classification (*p* = 0.001), N classification (*p* < 0.001), M classification (*p* < 0.001) and pathologic differentiation (*p* = 0.002). Patients with lower MAGED1 expression had a shorter survival time than those with higher MAGED1 expression. Univariate and multivariate analyses indicated that MAGED1 expression was an independent prognostic factors (*p* < 0.001).

**Conclusions:**

MAGED1 may serve as a novel prognostic biomarker of human colorectal cancer.

## Background

The melanoma antigen (MAGE) family, which includes more than 25 members, is classified into two subfamilies based on the structural differences of the genes and tissue-specific gene expressions. Type I *MAGE* genes are classically subdivided into three clusters (*MAGE-A*, *B*, and *C*), which are expressed in a variety of cancer cells, but are seldom expressed in normal cells [1-4]. Type II *MAGE* genes include *MAGE-D* (*MAGED1* to *MAGED14*), *MAGEE1* to *H1*, *MAGEL2* and *NECDIN*[5]. In contrast to type I *MAGE* genes, type II *MAGE* genes are expressed in a variety of normal tissues and cell lines [6,7].

Melanoma antigen D1 (MAGED1), also known as Dlxin-1 or NRAGE, is a member of the type II MAGE family. It was reported that MAGED1 modulated the transcriptional activity of DLx5/Msx2, regulating osteoblast differentiation during development [8,9]. Unlike the type I *MAGE* genes, which encode tumor antigens, *MAGED1* encodes a protein involved in the apoptosis pathway. MAGED1 mediates cellular apoptosis and cell cycle arrest through the c-JNK and p53-dependent pathways [10-12], and is also involved in the BRCA2-mediated cell proliferation arrest in a p53-independent manner [13].

In addition to normal tissue expression, type II *MAGE* genes, including *MAGED1*, were also detected in cancer cells. It was reported that the expression of MAGED1 was down-regulated in breast carcinoma cell lines [13] and in glioma stem cells [14]. Chung *et al.* examined the expression profile of MAGE family genes in Taiwanese patients with colorectal cancer and discovered that the type II *MAGE* genes *MAGED12*, *MAGEF1*, and *MAGEH1* are frequently up-regulated in tumors [15].

Although MAGED1 may play an important role in apoptosis and anti-tumorigenesis, there are no reports on its clinical role in colorectal cancer. In this study, we investigated MAGED1 expression and its clinical significance in human colorectal cancer. We found that MAGED1 expression was significantly down-regulated in colorectal cancer tissues compared with their adjacent non-tumorous tissues (ANT) and was associated with the clinical features of colorectal cancer. MAGED1 may serve as a novel prognostic biomarker of human colorectal cancer.

## Methods

### Patient information and tissue specimens

This study was conducted on a total of 285 paraffin-embedded, archived CRC primary samples, which were histopathologically and clinically diagnosed at the Sun Yat-sen University Cancer Center from 1999 to 2007. The clinical and clinicopathological classification and stage were determined according to the American Joint Committee on Cancer (AJCC) TNM staging system. Each lesion was graded histologically according to the WHO classification criteria. Overall survival (OS) was defined as the interval between the date of surgery and date of death or the last known follow up. For the use of these clinical materials for research purposes, prior consent of the patients and approval from the Institutional Research Ethics Committee were obtained. Six pairs of colorectal cancer tissue specimens and corresponding adjacent non-tumorous specimens were obtained from patients with CRC who underwent surgical CRC tissue resection at Sun Yat-sen University Cancer Center. Written informed consent was obtained from each patient before surgery. All excised samples were obtained within 1 h after the operation from tumor tissues and corresponding adjacent non-tumorous specimens 5–10 cm from the tumor. For all excised tissues, half of each specimen was placed into liquid nitrogen until further analysis and the remainder was fixed by formalin processed for immunohistochemistry (IHC). The clinical information related to the 285 CRC samples is described in detail in Table 1.

**Table 1:** Clinical data of 285 samples of colorectal cancer

	**Number of cases (%)**
**Gender**	
Male	183 (64.2)
Female	102 (35.8)
**Age (years)**	
≤ 50	111 (38.9)
> 50	174 (61.1)
**Location**	
colon	133 (46.7)
rectal	152 (53.3)
**Clinical Stage**	
I	47 (16.5)
II	61 (21.4)
III	88 (30.9)
IV	89 (31.2)
**T classification**	
T1	20 (7.0)
T2	44 (15.4)
T3	124 (43.5)
T4	97 (34.0)
**N classification**	
N0	123 (43.2)
N1	90 (31.6)
N2	72 (25.3)
**M classification**	
M0	196 (68.8)
M1	89 (31.2)
**Pathologic Differentiation**	
Poor	58 (20.4)
Moderate	205 (71.9)
Well	22 (7.7)
**Histological Types**	
Non-mucinous adenocarcinoma	268 (94.0)
mucinous adenocarcinoma	17 (6.0)
**Vital status (at follow-up)**	
Alive	173 (60.7)
Death (All colorectal cancer-related)	112 (39.3)
Low expression	161 (56.5)
High expression	124 (43.5)

### RNA extraction, reverse transcription and real-time PCR

Total RNAs from 6 pairs of tumor tissues and non-tumorous tissues was extracted using Trizol reagent (Invitrogen, Carlsbad, California, USA) according to the manufacturer’s instructions. First-strand cDNA was synthesized by reverse transcriptase (Invitrogen, Carlsbad, California, USA) using total RNA as a template. Real-time PCR was carried out using an ABI Prism 7500 Sequence Detection System (Applied Biosystems, Foster City, California, USA). The sequences of the primers were as follows: MAGED1: sense primer, 5′ GATTCCCTCAGACCTTTGC; anti-sense primer, 5′ GAAGGAATCTGAGGCTTCAG; 18S was amplified as an internal control using the following primers: sense primer, 5′ CCTGGATACCGCAGCTAGGA; anti-sense primer, 5′ GCGGCGCAATACGAATGCCCC. Real-time PCR was performed using programmed parameters for the SYBR Green method (Invitrogen, Carlsbad, California, USA) to collect the fluorescent signals, heating at 95°C for 5 min, followed by 95°C for 15 s, 60°C for 15 s and 72°C for 32 s for 40 cycles. All gene expression values were normalized using the housekeeping gene 18S and calculated using the comparative C_T_ method (ΔΔC_T_ method).

### Western blotting

Western blotting was performed according to standard methods as described previously [16]. MAGED1 expression was determined with anti-rabbit immunoglobulin G (1:2,000; Abcam, Cambridge, MA) according to the manufacturer’s suggested protocols. An anti-αtubulin mouse monoclonal antibody (1:2,000; Boster, Wuhan, China) was used as the loading control.

### Immunohistochemistry (IHC)

Immunohistochemistry was performed to study altered protein expression in 285 human colorectal cancer tissues. IHC was carried out according to standard methods as described previously [16]. Briefly, the tissue sections were deparaffinized in xylene at 37°C for 20 min and rehydrated. Endogenous peroxide was blocked by incubating the sections with 3% hydrogen peroxide in methanol for 20 min at 37°C. The sections were then submerged in 10 mM citrate buffer (pH 6.0) and microwaved for antigenic retrieval, followed by incubation with rabbit anti-MAGED1 (1:200; Millipore, Billerica, MA) overnight at 4°C. After washing, tissue sections were treated with anti-rabbit secondary antibody for 30 min, followed by further incubation with streptavidin horseradish peroxidase complex. The sections were developed with diaminobenzidine tetrahydrochloride (DAB) and counterstained with hematoxylin.

The proportion of the stained cells and the extent of the staining were used as criteria of evaluation. Slides were scored by two independent observers who were blinded to the patient data. For each case, at least 1,000 tumor cells were analyzed, and the percentage of positively stained tumor cells was recorded. For each sample, the proportion of MAGED1-expressing cells varied from 0% to 100%, and the intensity of staining varied from weak to strong. One score was given according to the percent of positive cells as follows: ≤ 10% = 0, >10% to ≤ 25% = 1, >25% to ≤ 50% = 2, >50% to ≤ 75% = 3, >75% = 4. Another score was given according to the intensity of staining as negative = 0, weak = 1, moderate = 2, or strong = 3. A final score was then calculated by multiplying the two above scores. If the final score was equal to or less than four, the tumor was considered as having low expression; otherwise, the tumor was considered as having high expression.

### Statistical analysis

All statistical analyses were performed using by the SPSS 16.0 statistical software package (SPSS Inc., Chicago, IL). The relationship between MAGED1 expression and the clinicopathologic characteristics was analyzed by the *χ*^2^ test. Survival curves were plotted by the Kaplan-Meier method and compared using the log-rank test. Survival data were evaluated using univariate and multivariate Cox regression analyses. A *p*-value of less than 0.05 was considered statistically significant in all cases.

## Results

### MAGED1 expression in paired colorectal cancer and non-tumorous tissues

**Figure 1:**
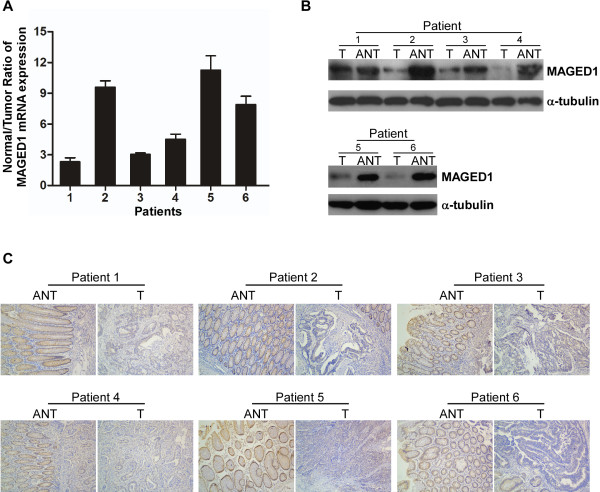
**Decreased expression of MAGED1-1 in colorectal cancer.A**, Real time RT-PCR analysis of the normal/tumor ratio of MAGED1-1 mRNA expression in each of the primary colon cancer tissues (T) and adjacent non-cancerous colon tissues (ANT) paired from the same patient. B-C, Western blotting (**B**) and IHC (**C**) analyses of MAGED1-1 protein in each of the primary colon cancer tissues (T) and adjacent non-cancerous colon tissues (ANT) paired from the same patient.

Real-time PCR, western blotting and IHC analysis showed that MAGED1 mRNA and protein expression were significantly down-regulated in all six pairs of human colorectal cancer tissues compared with matched adjacent non-tumorous tissues (Figure 1A-C) Additional file 1: Table S1.

131 colorectal cancer and matched ANT samples derived from the 285 archival primary colorectal cancer tissues were evaluated MAGED1 protein expression by IHC analysis Additional file 2: Table S2. We defined the scores less than or equal to four, including non-expression as low MAGED1 expression referring to their MAGED1 expression scoring system in the IHC samples, otherwise, they were considered as having high MAGED1 expression. According to the definition, the rate of low MAGED1 expression (74/131, 56.5%) in colorectal cancer samples significantly differed from the rate in matched ANT samples (13/131, 10.0%) (p = 0.031). Furthermore, MAGED1 expression was down-regulated in 58.8% (77/131) and up-regulated only in 22.1% (29/131) colorectal cancer tissues, compared with their paired ANT tissues according to the scoring system. These results suggest that MAGED1 expression is down-regulated in colorectal cancer tissues.

### Correlation between MAGED1 protein expression and clinicopathological features

MAGED1 protein expression was evaluated by immunohistochemistry in 285 paraffin-embedded, archival primary colorectal cancer tissues. The samples included 47 cases of clinical stage I (16.5%), 61 cases of stage II (21.4%), 88 cases of stage III (30.9%) and 89 cases of stage IV (31.2%) colorectal cancer. MAGED1 protein was detected in 261 of 285 CRC cases (91.6%), but in only 5 of 17 colorectal mucinous adenocarcinoma cases (29.4%). According to the scoring system, low MAGED1 expression was detected in 161/285 (56.5%) colorectal carcinomas, while the high MAGED1 expression was detected in 124/285 (43.5%).

As shown in Table 2, the relationship between the MAGED1 expression and clinical characteristics was analyzed in 285 CRC cases. There was no significant correlation between MAGED1 protein expression and gender, age, tumor location, or histological types of CRC. However, MAGED1 expression was closely associated with clinical stage (*p* < 0.001), T classification (*p* = 0.001), N classification (*p* < 0.001), M classification (*p* < 0.001) and pathologic differentiation (*p* = 0.002).

**Table 2:** Correlation between MAGED1-1 expression and clinicopathological characteristics of colorectal cancer patients

**Characteristics**		**MAGED1-1**		**Chi-square test *P*-value**
		**Low or none No. cases (%)**	**High No. cases (%)**	
Gender	Female	63 (39.1)	39 (31.5)	0.213
	Male	98 (60.9)	85 (68.5)	
Age (years)	≤ 50	65 (40.4)	46 (37.1)	0.625
	> 50	96 (59.6)	78 (62.9)	
Location	colon	70 (43.5)	63 (50.8)	0.233
	rectal	91 (56.5)	61 (49.2)	
Clinical Stage	I + II	35 (21.7)	73 (58.9)	**<0.001**
	III	60 (68.2)	28 (31.8)	
	IV	66(74.2)	23(25.8)	
T classification	T1 + T2	24 (14.9)	40 (32.3)	**0.001**
	T3 + T4	137 (85.1)	84 (67.4)	
N classification	No	43 (26.7)	80 (64.5)	**<0.001**
	Yes	118 (73.3)	44 (35.5)	
M classification	M0	95 (59.0)	101 (81.5)	**<0.001**
	M1	66 (41.0)	23 (18.5)	
Pathologic Differentiation	Poor	44 (27.3)	14 (11.3)	**0.002**
	Moderate	108 (67.1)	97 (78.2)	
	Well	9 (5.6)	13 (10.5)	
Histological Types	Non-mucinous adenocarcinoma	148 (92.5)	120 (96.0)	0.227
	mucinous adenocarcinoma	12 (7.5)	5(4.0)	

**Figure 2:**
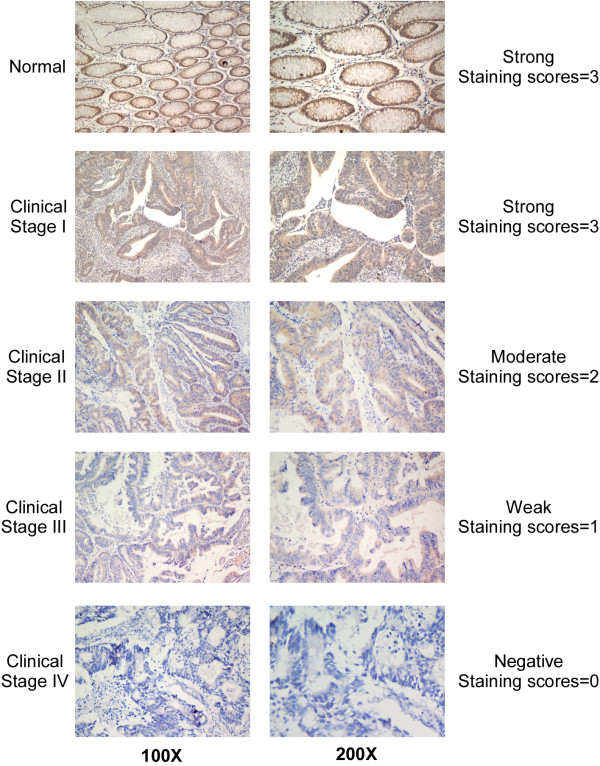
**Decreased expression of MAGED1-1 in advanced colorectal cancer.** Representative IHC analyses of MAGED1-1 expression in normal colorectal tissues and colorectal cancer specimens of different clinical stages (left column). The examples of the scoring system for different scores were also showed (right column).

**Figure 3:**
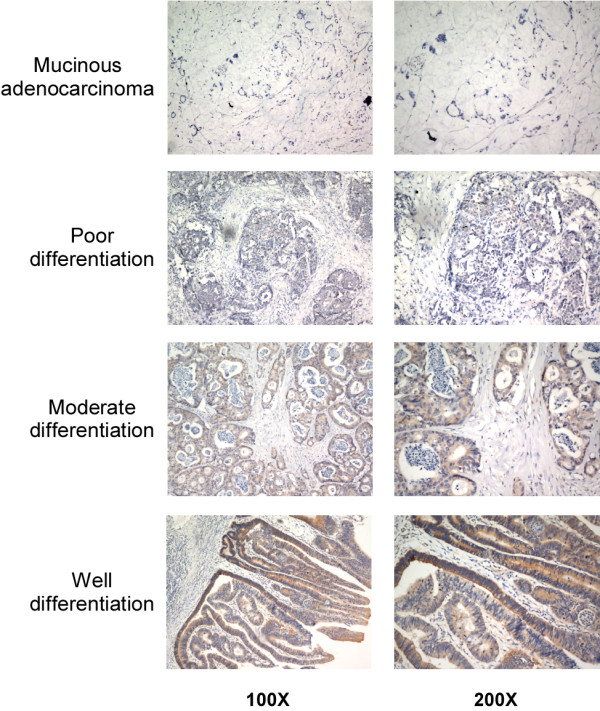
MAGED1-1 expression in colorectal cancer tissues with different pathologic differentiation.

The MAGED1 protein expression was inversely correlated with clinical stage and T classification. Higher staging and poor pathological differentiation were correlated with lower MAGED1 expression (Figures 2 and 3). In addition, most of the colorectal mucinous adenocarcinoma cases (12/17) were demonstrated low MAGED1 expression.

### Survival analysis

**Figure 4:**
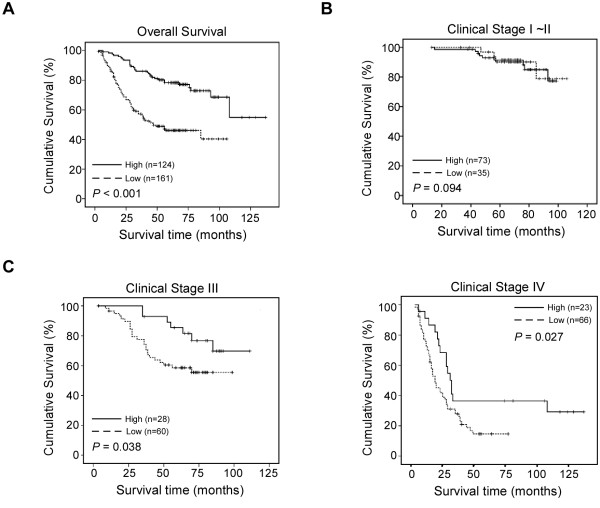
**Kaplan-Meier curves with univariate analyses (log-rank) for patients with low MAGED1-1 expression (dotted line) versus high MAGED1-1 expression tumors (bold line).A**, The overall survival of patients (clinical stages I-IV) with low/high MAGED1-1 expression. **B**, The overall survival of patients (clinical stages I-II) with low/high MAGED1-1 expression. **C**, The overall survival of patients (clinical stages III and IV) with low/high MAGED1-1 expression.

A Kaplan-Meier analysis and the log-rank test were used to calculate the effects of the clinicopathological characteristics and MAGED1 expression on survival. The expression of MAGED1 in colorectal cancer was significantly correlated with patients’ survival time (*p* <0.001). Patients with lower MAGED1 expression had a shorter overall survival time (OS) than those with higher MAGED1 expression (median OS 47 months vs has not been reached, respectively; p < 0.001). The overall two-, three-, and five-year accumulative survival rates were 68.8%, 57.2%, and 46.1%, respectively, in cases with low MAGED1 expression and were 93.5%, 80.2%, and 78.4%, respectively, in cases with high level of MAGED1 expression(Figure 4A). Additionally, similar results were obtained in stage III and IV subgroup patients (Figure 4C), but stage I-II subgroup patients did not show the similar results (Figure 4B).

Furthermore, univariate and multivariate analyses indicated that clinical stage, pathologic differentiation, and MAGED1 expression were independent prognostic factors (Table 3), suggesting that MAGED1 may be a prognostic factor for survival in colorectal cancer patients.

**Table 3:** Univariate and multivariate analysis of various prognostic parameters in patients with colorectal cancer Cox-regression analysis

**Variables**	**Univariate analysis**		**Multivariate analysis**		
	**No.**	***P *value**	**Hazard Ratio**	**95% CI**	***P *value**
MAGED1-1		**<0.001**	0.473	0.305-0.734	**0.001**
low expression	161				
high expression	124				
Age		0.388	1.006	0.992-1.020	0.419
≤ 50	111				
> 50	174				
Gender		0.555	1.048	0.706-1.555	0.817
Male	183				
Female	102				
Differentiation		**0.012**	0.667	0.460-0.965	**0.032**
Poor	58				
Moderate	205				
Well	22				
Clinical stage		**<0.001**	3.127	2.404-4.068	**<0.001**
I	47				
II	61				
III	88				
IV	89				

## Discussion

In the present study, we demonstrated that MAGED1 expression was down-regulated at both the mRNA and protein levels in colorectal cancer tissues compared to matched adjacent non-tumorous tissues. Low levels of MAGED1 expression were more frequently observed in CRC patients with poor pathologic differentiation or those with advanced stages. This is the first study to analyze the prognostic relevance of the MAGED1 expression in colorectal carcinoma. We demonstrated that high MAGED1 expression was correlated with a better survival outcome, whereas low MAGED1 expression was correlated with a poorer survival outcome. Furthermore, MAGED1 expression was an independent prognostic factor, suggesting that MAGED1 may be a prognostic factor for survival in colorectal cancer patients.

MAGED1 expression may also be associated with the histological types in CRC. We found that MAGED1 expression was low in most of the mucinous adenocarcinomas of CRC (12/17). Conversely, the rate of low MAGED1 expression (148/268) in non-mucinous adenocarcinoma did not significantly differ from the rate of high expression (120/268). However, because we could only obtain a small number of mucinous adenocarcinoma samples, we were unable to demonstrate a significant correlation between the MAGED1 expression and the histological types in CRC (*p* = 0.227). The inclusion of a greater number of mucinous adenocarcinoma samples may resolve the problem.

We also failed to observe a significant relationship between the MAGED1 expression and CRC patients’ survival in the clinical stages I ~ II. We believe that this is due to the good prognosis of the early stage CRC patients and limit number of the clinical cases. However, there were significant correlations between the MAGED1 expression and overall survival in all patients and in clinical stage III and IV patients.

MAGED1 expression was also evaluated by Chung *et al.* in Taiwanese CRC patients, and reported MAGED1 overexpression occurred in 45% CRC patients [15]. In the present study, 131 CRC patients were enrolled to compare their MAGED1 expression between colorectal cancer tissues and paired adjacent non-tumorous tissues. The MAGED1 expression was down-regulated in 58.8% (77/131) and up-regulated only in 22.1% (29/131) CRC patients. Compared the patients’ clinical characteristics in these two studies, we found that the stage IV patients were 6.0% (6/100) vs 26.0% (34/131) in Chung *et al.*’s and our study, respectively. Importantly, the present study has shown that higher staging was correlated with lower MAGED1 expression. Thus, we deduce that the lower MAGED1 overexpression rate in our study was most probably because of the different distribution of clinical stages in patients. On the other hand, different research designs were performed in these two projects. Chung *et al.*’s study was detected MAGED1 expression on gene level; whereas our research was focus on its expression on protein level, which post-translational modifications may be involved in the expression regulation.

Different from the MAGED1, MAGED12 was reported frequently up-regulated in tumors [15,17]. It was reported that *MAGED1* and *-D2* RNA had different distribution during the embryonic development and brain development [18]. All these data suggested that different types of MAGE genes may play distinct roles in biochemical activities.

A circadian rhythm is an approximate 24-h period in the biological process of living entities, controlled by endogenous clock genes [19,20]. Clock genes include *period* (*per*), *clock* (*clk*), *Bmal1*, *Rev-erb α*, *cryptochrome (cry)*, and others [21]. MAGED1 was reported to regulate the expression of Bmal1, Rev-erb α, and E4bp4 by binding to the RORα protein. The depletion of MAGED1 *in vivo* has been shown to cause severely dampened oscillations of Bmal1 mRNA expression, resulting in an increased the clock speed [22].

Mounting evidence shows that circadian disruption increases cancer incidence and the cancer growth rate, suggesting that circadian genes participate in the growth and development of various cancers. Per2-deficient mice showed a marked increase in tumor development and reduced apoptosis in thymocytes following γ-radiation [23]. Alternatively, overexpression of Per2 inhibited tumor proliferation *in vitro*[24] and *in vivo*[25]. Other clock genes, such as *Bmal1*, *Clock*, *Cry* and *Rev-erbα*, have also been correlated with cancer [26-29]. In the present study, we demonstrated that MAGED1 also has a close relationship with the clinical features of colorectal cancer, with higher MAGED1 expression in CRC patients correlating with better survival and vice versa. Because MAGED1 regulates Bmal1 and Rev-erb α expression and dampens the oscillations of Bmal1 expression, MAGED1 depletion can induce circadian rhythm disorders [22]. We hypothesize that this may be the mechanism by which MAGED1 expression correlates with the CRC patients’ clinical features. However, the precise mechanism of the MAGED1 involvement in CRC development is still unclear. Thus, the further study including overexpression and knockdown of MAGED1 expression in CRC cells will be needed to explore the mechanism by which MAGED1 is involved in the development and progression of colorectal cancer and its exact regulating pathway *in vitro* and *in vivo*.

## Conclusion

In the present study, we found that MAGED1 expression was significantly down-regulated in colorectal cancer tissues compared with adjacent non-tumorous tissues and was associated with clinical stage, T classification, N classification, M classification and pathologic differentiation. MAGED1 expression was significantly correlated with overall survival in colorectal cancer patients. Patients with lower MAGED1 expression had a shorter survival time than those with higher MAGED1 expression. MAGED1 may serve as a novel prognostic biomarker of human colorectal cancer.

## Abbreviations

AJCC: American Joint Committee on Cancer; c-JNK: c-Jun N-terminal kinase; CRC: Colorectal cancer; DAB: Diaminobenzidine tetrahydrochloride; IHC: Immunohistochemistry; ANT: Non-tumorous tissues; MAGED1: Melanoma antigen D1; MAGE: Melanoma antigen; OS: Overall survivalx; qPCR: Quantitative real time polymerase chain reaction; SCN: Suprachiasmatic nucleusx.

## Misc

Zhao-lei Zeng and Wen-jing Wu are contributed equally to this work.

## Competing interests

We have no financial or personal relationships with other people or organizations that would bias our work. No benefits in any form have been received or will be received from a commercial party related directly or indirectly to the subject of our article.

## Authors’ contributions

ZZL conceived the study, carried out the IHC and drafted the manuscript. WWJ carried out the IHC and WB, participated in the clinical data collection of the colorectal carcinoma patients. YJ carried out the qPCR. TZJ, CDL, QMZ, LHY and WZQ participated in the clinical data collection. JY and WDS performed the statistical analysis. XRH conceived the study, participated in its design and final approval of the version to be published. All authors read and approved the final manuscript.

## Supplementary Material

Additional file 1 **Table S1.** Clinical data of 6 patients of colorectal cancer in Figure 1. Click here for file

Additional file 2 **Table S2.** Clinical data of 131 CRC patients compared their MAGED1 expression between the colorectal cancer tissues and the paired adjacent normal tissues. Click here for file
