# Supplementary material for: Prognostic relevance of melanoma antigen D1 expression in colorectal carcinoma
Source: J Transl Med. 2012 Aug 31;10:181. doi: 10.1186/1479-5876-10-181 (PMC3494540; doi:10.1186/1479-5876-10-181)
Supplement: Additional file 1 — Table S1. Clinical data of 6 patients of colorectal cancer in Figure 1. [file 1479-5876-10-181-S1.doc]

**Additional file 1: Table S1. Clinical data of 6 patients of colorectal cancer in Figure 1**

|  |  | Number of cases (%) |
| --- | --- | --- |
| **Gender** |  |  |
| Male |  | 4(66.7) |
| Female |  | 2(33.3) |
| **Age (years)** |  |  |
| ≤ 50 |  | 1(16.7) |
| > 50 |  | 5(83.3) |
| **Clinical Stage** |  |  |
| I |  | 0 |
| II |  | 2(33.3) |
| III |  | 4(66.7) |
| IV |  | 0 |
| **T classification** |  |  |
| T1 |  | 0 |
| T2 |  | 0 |
| T3 |  | 6(100) |
| T4 |  | 0 |
| **N classification** |  |  |
| N0 |  | 2(33.3) |
| N1 |  | 2(33.3) |
| N2 |  | 2(33.3) |
| **M classification** |  |  |
| M0 |  | 6(100) |
| M1 |  | 0 |
| **Pathologic Differentiation** |  |  |
| Poor |  | 1(16.7) |
| Moderate |  | 5(83.3) |
| Well |  | 0 |
