# Supplementary material for: Prognostic relevance of melanoma antigen D1 expression in colorectal carcinoma
Source: J Transl Med. 2012 Aug 31;10:181. doi: 10.1186/1479-5876-10-181 (PMC3494540; doi:10.1186/1479-5876-10-181)
Supplement: Additional file 2 — Table S2. Clinical data of 131 CRC patients compared their MAGED1 expression between the colorectal cancer tissues and the paired adjacent normal tissues. [file 1479-5876-10-181-S2.doc]

**Additional file 2: Table S2. Clinical data of 131 CRC patients compared their MAGED1 expression between the colorectal cancer tissues and the paired adjacent normal tissues**

|  |  | Number of cases (%) |
| --- | --- | --- |
| **Gender** |  |  |
| Male |  | 85(64.9) |
| Female |  | 46(35.1) |
| **Age (years)** |  |  |
| ≤ 50 |  | 52(39.7) |
| > 50 |  | 79(60.3) |
| **Clinical Stage** |  |  |
| I |  | 26(19.8) |
| II |  | 33(25.2) |
| III |  | 38(29.0) |
| IV |  | 34(26.0) |
| **T classification** |  |  |
| T1 |  | 8(6.1) |
| T2 |  | 21(16.0) |
| T3 |  | 57(43.5) |
| T4 |  | 45(34.4) |
| **N classification** |  |  |
| N0 |  | 63(48.1) |
| N1 |  | 38(29.0) |
| N2 |  | 30(22.9) |
| **M classification** |  |  |
| M0 |  | 96(73.3) |
| M1 |  | 35(26.7) |
| **Pathologic Differentiation** |  |  |
| Poor |  | 28(21.4) |
| Moderate |  | 98(74.8) |
| Well |  | 5(3.8) |
